# A *de novo CTNNB1* nonsense mutation associated with syndromic atypical hyperekplexia, microcephaly and intellectual disability: a case report

**DOI:** 10.1186/s12883-016-0554-y

**Published:** 2016-03-12

**Authors:** Anna Winczewska-Wiktor, Magdalena Badura-Stronka, Anna Monies-Nowicka, Michal Maciej Nowicki, Barbara Steinborn, Anna Latos-Bieleńska, Dorota Monies

**Affiliations:** Chair and Department of Child Neurology, Poznan University of Medical Sciences, Poznan, Poland; Chair and Department of Medical Genetics, Poznan University of Medical Sciences, ul. Rokietnicka 8, 60-608 Poznań, Poland; Poznan University of Medical Sciences, Poznan, Poland; Department of Genetics, King Faisal Hospital and Research Centre, Riyadh, Saudi Arabia

**Keywords:** β-catenin, Hyperekplexia, Microcephaly, Intellectual disability

## Abstract

**Background:**

In addition to its role in cell adhesion and gene expression in the canonical Wingless/integrated Wnt signaling pathway, β-catenin also regulates genes that underlie the transmission of nerve impulses. Mutations of *CTNNB1* (β-catenin) have recently been described in patients with a wide range of neurodevelopmental disorders (intellectual disability, microcephaly and other syndromic features). We for the first time associate *CTNNB1* mutation with hyperekplexia identifying it as an additional candidate for consideration in patients with startle syndrome.

**Case presentation:**

We describe an 11 year old male Polish patient with a *de novo* nonsense mutation in *CTNNB1* who in addition to the major features of *CTNNB1*-related syndrome including intellectual disability and microcephaly, exhibited hyperekplexia and apraxia of upward gaze. The patient became symptomatic at the age of 20 months exhibiting delayed speech and psychomotor development. Social and emotional development was normal but mild hyperactivity was noted. Episodic falls when startled by noise or touch were observed from the age of 8.5 years, progressively increasing but never with loss of consciousness. Targeted gene panel next generation sequencing (NGS) and patient-parents trio analysis revealed a heterozygous *de novo* nonsense mutation in exon 3 of *CTNNB1* identifying a novel association of β-catenin with hyperekplexia.

**Conclusion:**

We report for the first time a clear association of mutation in *CTNNB1* with an atypical syndromic heperekplexia expanding the phenotype of *CTNNB1*-related syndrome. Consequently *CTNNB1* should be added to the growing list of genes to be considered as a cause of startle disease or syndromic hyperekplexia.

## Background

A novel dominant intellectual disability (ID) syndrome caused by β-catenin gene (*CTNNB1)* haploinsufficiency was recently identified [1]. Mutations in *CTNNB1* (chr3: 41,194,837-41,260,096) are responsible for a wide spectrum of neurodevelopmental disorders. The phenotypes of all 21 patients with *CTNNB1* mutations reported to date in four different studies are broad and consistently include several major features: ID and motor delay with different degrees of severity, speech impairment, behavioral anomalies, spastic paraplegia, microcephaly and overlapping facial features [1–4]. In addition, variable clinical findings may involve brain MRI abnormalities (corpus callosum thinning and enlarged ventricles) and spinal anomalies (syringomyelia or tethered cord) [2]. We report a case of an 11-year-old boy with a *de novo* nonsense mutation in *CTNNB1* who presented with mild ID, ataxia, spastic paraplegia, mild microcephaly and dysmorphic features. Notably our patient exhibited hyperekplexia and apraxia of upward gaze, features which to date have not been described in patients with mutations of *CTNNB1*.

**Table 1:** Clinical findings in patients with mutation in the N-terminal of β-catenin. * Previously published patients [2], n.e. not examined, n.r. not recorded

	Our Patient	Patient 5*	Patient 10*	Patient 13*
*Gender*	M	F	F	M
*Gestational weeks*	39	40	40	39
*Apgar scores*	10	9/10/10	n.r.	10/10
*Birth weight [g]*	2900	3450	3300	3160
*Birth length [cm]*	53	51	50.8	49
*Age at last examination [y]*	11	5 7/12	4	5 6/12
*Height [cm]*	150	108	104.5	112
*Weight [kg]*	46	15	14.15	17.2
*BMI [kg/m2]*	20.4	12.9	13.0	13.7
*OFC [cm]*	51	47.3	46	48.5
*Microcephaly*	+	+	+	+
*Croniofacial dysmorphism*				
*broad nasal tip*	+	+	+	(+)
*small alae nasi*	+	+	+	-
*long and/or flat philtrum*	−/+	+/+	+/+	−/+
*thin upper lip vermillion*	+	+	+	+
Developmental and neurological abnormalities				
*Truncal hypotonia*	+	+	+	+
Peripheral hypertonia/spasticity	+	+ atactic gait	+	+ legs > arms
Motor delay Crowling (months) Free walking	moderate	severe	moderate	moderate
	did not crawl	24	12	25
	3 years	not yet, walking frame	4 years, falls a lot	not yet, walking frame
Speech impairment	moderate, unclear speech	severe, few words (30–34), sign language	speech apraxia, ~50 words	short sentences at 6 years
Basic speech comprehension	+	+	+	+ good
Intellectual disability	+	+	+	+ (no formal test available)
*Regression*	+ speech	?	-	-
*Behavioral characteristics*	temper tantrums/crying, friendly personality, aggression, frustration, anxiety, sleep disturbances, stereotypic movements	sleep disturbances, stereotypic movements, autoagression	normal	normal, friendly, sensitive to loud noises
*Seizures*	-	-	-	-
*EEG*	during episodes diffused fast background activity	normal	normal	n.e.
*Brain MRI*	arachnoid cyst, enlarged Sylvius sulcus	normal	basically normal	normal
*Hearing loss*	-	-	-	-
*Vision*	hyperopia, astigmatism, strabismus	hyperopia, intermittent strabismus	normal	strabismus
*Others*				
*Internal abnormalities*	-	-	-	-
*Frequent infections*	-	-	-	+ (infancy)
*Miscellaneous*	ginecomastia, inverted nipples	-	feeding difficulties	-
*Hands*	slender and long fingers	thin fingers, short distal phalanges	normal	clinodactyly V, hand and feet
*Feet*	sandal gap, long toes	flat feet	normal	pes equines

## Case Presentation

The study was approved by an ethics committee of Poznan University of Medical Sciences, Poland. Parents provided written informed consent according to the Declaration of Helsinki for publication of clinical information. The index case, a male patient was born at term as the first child to healthy, young, non-consanguineous Caucasian parents. Family history was negative for the presence of neurological disorders. He weighed 2900 g, with a good Apgar score. After a normal neonatal period, delayed psychomotor and speech development was evident (at 20 months of age he was unable to walk unassisted and was able to speak only a few words). His social and emotional development was within normal range but hyperactivity was observed. No focal lesions were visualized in 1.5 T MRI; notably in cortex, thalami, midbrain and medulla, except for a 12–13 mm in diameter arachnoid cyst in the posterior fossa and an enlarged Sylvian fissure. An EEG recording showed mild slowing of background activity. The parents had noticed episodic falls since the age 8.5 years. The episodes were provoked by noise, touch or rapid movement in the neighbourhood. Sudden loss in muscle tonus of lower limbs was observed, together with an increase in axial muscle tonus. It led to a rapid flexion in knees and hips provoking a fall. The boy has never had any head injury while falling, due probably to the extension position of his trunk. Episodes were short, lasting a second or two, without loss of consciousness. The boy was able to lift up by himself very quickly directly afterwards and was able to recall the entire episode. This sequence was highly repetitive. A progressive increase in the frequency of falls was noticed. Neurological examination between episodes indicated a left hemiparesis with positive Babinski sign and mild dysarthria. EEG during episodes showed diffused fast background activity, typical for hyperekplexia. In the interictal EEG no epileptiform discharges were observed. Levetiracetam was introduced with good clinical response however the drug was withdrawn because of behavioral disturbances (aggression, tendency to cry). Brivaricetam was introduced instead and the effect was similar to Levetiracetam with fewer side effects. During the last examination at the age of 11 years problems with speech were shown to have increased and coordination problems appeared. In addition to episodes of hyperekplexia, he presents with mild ID, ataxia, truncal hypotonia, bilateral spastic paraplegia, dysarthria and apraxia of upward gaze. He needs support to walk. Ophthalmic examination showed astigmatism and hyperopia with a normal fundus of the eye. Periodical horizontal nystagmus is present but vision evoked potentials are normal. The boy has height and weight within normal ranges, he is mildly dysmorphic (asymmetric face, right frontal hair upsweep, hypotelorism, deep set eyes, strabismus, diastema, small teeth, short philtrum, thin upper lip vermillion, prominent columella, broad nasal tip, small alae nasi, hypoplastic upper crus of the inner helix), has long slender fingers and toes, mild scoliosis, gynecomastia, inverted nipples and microcephaly. He is generally a friendly child, rather anxious, avoiding eye contact, with episodes of frustration. Verbal aggression, crying and sleep disturbances have been also observed (Table 1).

**Fig. 1:**
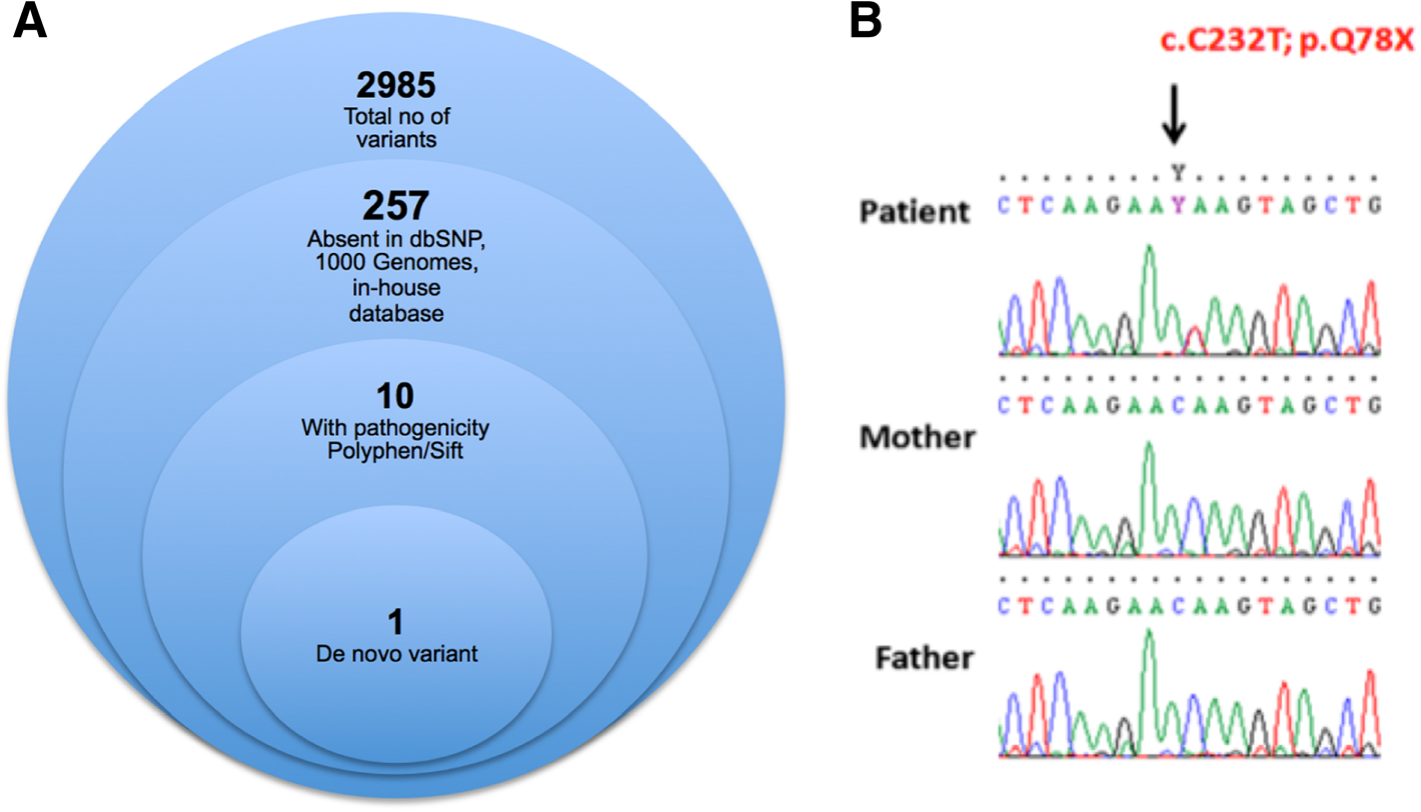
Filtering strategy used for identification of a causative mutation using trio analysis and NGS-gene panel (**a**). DNA electrophoregram with the c.C232T; p.Q78X mutation in exon 3 of *CTNNB1* (**b**)

The patient was followed by neuropediatrics and based upon symptoms including ataxia with progressive pyramidal syndrome, combined with supranuclear vertical gaze palsy, Niemann-Pick type C disease was assumed initially. However, sequencing of the entire coding and flanking regions of *NPC1* and *NPC2* failed to identify any pathogenic mutations. We further analyzed the index case using a NGS gene panel (targeted resequencing) that included 758 OMIM genes (including *GLRA1*, *GLRB* and *SLCA*) associated with neurological disorders (genomebiology.com/content/supplementary/s13059-015-0693-2-s4.xls). Genes were amplified and a library constructed using an AmpliSeq HiFi mix, proprietary primers and library kit (Thermo Fisher, Carlsband, CA, USA) followed by sequencing on the Ion Proton platform following the manufacturer’s protocol (Thermo Fisher, Carlsband, CA, USA). Sequences were mapped to hg19 and variants were called and annotated using the ion torrent pipeline (Thermo Fisher, Carlsband, CA, USA). Sequencing identified 2985 variants relative to hg19. By excluding previously reported variants (present in RefSeq, OMIM, Genbank, dbSNP, 1000 genomes, in-house database) and only focusing on non-synonymous, splicing, frameshift, indel and nonsense variants we decreased the number to 10 heterozygous changes each in a different gene (Fig. 1A). All changes were validated by Sanger sequencing of the index case and parents. Nine changes were inherited with the only other variant being confirmed as a heterozygous *de novo* nonsense mutation in exon 3 of *CTNNB1* (NM_001098209): c.C232T; p.Q78X (Fig. 1B).

## Conclusions

In this study we present an 11 year old boy in which we detected a *de novo* nonsense mutation in β-catenin (*CTNNB1).* Mutations of *CTNNB1* were recently reported to be associated with a rare ID syndrome with consistent clinical features including significant motor delay with hypotonia of the trunk, progressive distal hypertonia/spasticity of the legs, speech impairment, behavioral anomalies, frequently microcephaly and overlapping dysmorphic facial features [1–4]. Our patient’s phenotype was consistent with, albeit generally milder than major signs of this entity. However, the boy also suffers from hyperekplexia and apraxia of upward gaze. These features have not been previously described in patients with a mutation of *CTNNB1*.

Hyperekplexia, or human startle disease, (OMIM#149400) is a rare, nonepileptic, paroxysmal neurogenetic disorder characterized by hypertonia and exaggerated persistent startle reflex to unexpected auditory, visual, and somatosensorial stimuli [5, 6]. The cluster of these symptoms is associated with failure of glycinergic synaptic transmission often resulting from mutations in genes encoding presynaptic and postsynaptic proteins [7]. Hyperekplexia results from mutations in *GLRA1* and *GLRB* encoding the human glycine receptor and also the glycine transporter *SLCA5* [8, 9]. Later, mutations in the proteins (GPHN and ARHGEF9) that cluster and localize the inhibitory glycine and GABA receptors were identified [10, 11]. However, patients with defects in these synaptic clustering proteins presented a disorder that had only a degree of phenotypic overlap with hyperexplexia. The genetic basis of hyperekplexia in a large proportion of cases remains to be discovered [12]. In our case the clinical diagnosis of hyperekplexia is based on the presence of its typical features: falls as a reaction to sudden stimuli, paroxysmal character, shortness and repetitiveness of episodes (that differenciate hyperekplexia from episodic falls and dyskinesias), no epileptiform EEG discharges during episode nor loss of conciousness (that are observed in atonic seizures), rapid recovery (that is never seen in cataplexy). However, the hyperekplexia in our patient lacks some of cardinal features of hyperekplexia such as rigidity during first year of life, general increase of muscle tonus during an episode and frequent head injuries. Presence of progressive neurologic impairment is also not typical for hyperekplexia. Therefore, we propose a diagnosis of atypical hyperekplexia for this patient`s neurological condition.

β-catenin (CTNNB1) belongs to the armadillo family of proteins responsible for regulation of gene expression in the canonical Wnt signaling pathway, in addition to their function in cell adhesion [13]. Wnt family secreted glycoproteins are highly conserved and modulate cell-cell communication in cellular processes including central nervous system development [14]. A central component of this pathway, β-catenin binds the TCF/LEF transcription factors that are involved in regulation of many genes [15]. In the thalamus, β-catenin regulates the expression of genes encoding proteins associated with excitation of neurons. In the mouse, it binds to *Gabra 3* encoding the GABA receptor, and also to genes responsible for voltage and ligand-gated ion channels: *Cacna1g* and *Kcna6.* This indicates a role of β-catenin in the maintenance of neuronal excitability [13].

During axonal developmental, the specification of the axon initial segment (AIS), characterized by a high density of voltage-gated Na^+^ and K^+^ channels, is critical in initiating and modulating action potentials [16]. These ion channels are tethered by ankyrinG (intracellular protein) to the membrane of the AIS and during this process, β-catenin is progressively accumulated at the AIS and is an essential component of this neuronal domain [17]. Its enrichment plays a role in dendritic morphogenesis, axonal growth and the maturation of AIS functions, modulating neuronal excitability and voltage-gated sodium channels [13, 18]. β-catenin knockdown decreases voltage-gated sodium currents at the AIS. The sodium current reduction is most likely due to diminished ankyrinG tethering at the AIS and the loss of AIS integrity when β-catenin is absent [17]. This molecular mechanism underlying neuronal action potential generation explains β-catenin involvement in disorders related to neuronal development and excitability [17].

Our patient expresses hyperekplexia, a unique symptom that has not been seen among other reported *CTNNB1* patients. Only in one other case (P14) a pronounced startle response on both auditory and visual stimuli provoking a breath holding spell was noted without a clear diagnosis of hyperekplexia [2]. Our patient has a *de novo* mutation in *CTNNB1* (c.C232T; p.Q78X) which is localized in the N-terminal domain of β-catenin. Three other patients (P5, P10 and P13) described by Kuechler’s et al. also had mutations in the N-terminal helical domain [2]. This region contains a conserved short linear motif responsible for binding β-TrCPE3 ubiquitin ligase, but only when it is phosphorylated. Degradation of β-catenin is regulated by this N-terminal segment. When comparing the clinical findings for all four patients with mutation in the N-terminal of β-catenin, consistent phenotypic features emerge with exception of atypical hyperekplexia and impaired upward gaze unique to our case (Table 1). In some cases with *CTNNB1* haploinsufficiency syndrome, there are patients with additional features not seen in others [4]. There is also, perhaps not surprisingly, inconsistency in the phenotype resulting from heterozygous loss-of-function mouse and human mutations, with phenotype in humans being much more severe [3]. This wide spectrum of clinical features in cases with *CTNNB1* mutations may result from compensatory activity of other protein partners that can assume the role of β-catenin in its absence [3]. Plakoglobin for instance (also called γ-catenin) has a very similar structure and ligand binding capacity to β-catenin [19]. The data presented here provides new insight into the role of β-catenin in brain development and its association with neurodevelopmental disorders including hyperekplexia. *CTNNB1* should be considered a candidate in patients with hyperekplexia in whom classical genetic mechanisms identified to date are negative.

### Consent

Written informed consent was obtained from the patient’s parents for publication of this Case Report and any accompanying images. A copy of the written consent is available for review by the Editor-in-Chief of this journal.

## Abbreviations

Wnt: Wingless/Integrated
NGS: Next Generation Sequencing
CTNNB1: β-catenin protein
GABA: γ-aminobutyric acid
EEG: Electroencephalogram
TCF/LEF: T-cell factor-1 (Tcf-1) and lymphoid enhancing factor-1 (Lef-1)
